# Supplementary material for: The zipper mechanism in phagocytosis: energetic requirements and variability in phagocytic cup shape
Source: BMC Syst Biol. 2010 Nov 8;4:149. doi: 10.1186/1752-0509-4-149 (PMC2991294; doi:10.1186/1752-0509-4-149)
Supplement: Additional file 1 — Supplementary information. The supplementary information file contains additional discussion on parameter values in simulations, as well as additional simulation results and fluorescence images analysis. It includes 10 supplementary figures, each accompanied by a short descriptive paragraph. [file 1752-0509-4-149-S1.PDF]

The zipper mechanism in phagocytosis: energetic requirements and variability in phagocytic cup shape

Supplementary information

Sylvain Tollis[1,2,], Anna E Dart[2,3], George Tzircotis[2,3], and Robert G Endres[1,2,*]

[1]Division of Molecular Biosciences, Imperial College London
[2]Centre for Integrated Systems Biology at Imperial College
[3]Division of Cell and Molecular Biology, Imperial College London,
South Kensington campus, SW7 2AZ, London, United Kingdom

[*] To whom correspondence should be addressed. E-mail: r.endres@imperial.ac.uk

Contents

Additional discussion on parameter values and simulations 2
1 - Choosing model parameters 2
2 - Details of Monte Carlo algorithm 2

Additional simulations 3
3 - Ratchet model in action 3
4 - Role of engulfment kinetics in cup shape 3
5 - Influence of membrane fluctuation width on cup shape 5
6 - Reproductibility of cup shapes 5
7 - Active versus passive engulfment of large particles 5
8 - Stalling at partial engulfment 5

Additional analysis of fluorescence images 6
9 - Statistics of phagocytic cup shapes 6
10 - Average cup height versus percentage of engulfed particle surface area 7
11 - Alternative image analysis of phagocytic cup shapes 7

References 7

Additional discussion on parameter values and simulations

1 - Choosing model parameters

According to previous experimental studies of phagocytes, a cell's bulk bending rigidity κ_b may vary in a range from $0.03-2$ pNμm depending on the type of cell and measurement method used (see [1] and references therein). Similarly, the bulk surface tension σ may range from $2-4\times10^{-2}$ mNm^{-1} in resting neutrophils but can vary in other cell types [1–3]. However, during phagocytosis of large particles with radius 5μm by aspirated spherical neutrophils, an abrupt increase in surface tension was observed (to 0.8 mNm^{-1} [3]) during the uptake. Note also that, κ_b and σ may vary on the scale of the cell [4–6]. Changes in local phospholipid and protein composition of the cups membrane may influence κ_b [7]. Furthermore, during engulfment membrane delivery from internal buffers [8–11] and unfolding of surface membrane folds [12] may strongly reduce σ at the cup. Therefore in our simulations we use values of κ_b and σ slightly lower than previously cited values, *i.e.* $\kappa_b = 1.3\times10^{-2}$ pNμm and $\sigma = 6.2\times10^{-6}$ mNm^{-1} in order to produce realistic cup shapes.

The FcγR-IgG binding free energy ΔF_{LR} was measured to $16k_BT$ [13], $18-20k_BT$ [14, 15], and $23k_BT$ for IgE to Fcϵ RI binding [16] at physiological temperature. The density d_{LR} of IgG-FcγR bonds was estimated to range from $270-435$ μm^{-2} for macrophages [17]. The total binding energy density ϵ is consequently of the order of $\Delta F_{LR} d_{LR} = 30$ pNμm^{-1}. Note that due to simple diffusion or in response to signaling, additional receptors may be recruited to the phagocytic cup, which may lead to a higher binding energy density. Additionally, receptors may cluster, leading to a non-uniform ligand-receptor binding-energy density in cells. Taken together, we chose a ligand-receptor binding energy density $\epsilon = 58.5$ pNμm^{-1} slightly higher than measured in resting cells.

The cell-volume constraint was chosen $\kappa_P = 2.56\times10^{-5}$ pNμm^{-5}. This reflects strong regulation of cell volume on the one hand [18, 19], but, on the other hand, allows volume fluctuations of about 20%, in line with observed distributions of cell volumes [20, 21].

Finally, the algorithm convergence is maximized if approximately half of the trial moves are accepted. To satisfy this requirement without constraining the biophysical parameters, we have modulated the trial moves acceptance rate by choosing an intermediate cutoff $w_{\max}$ for the width of the membrane fluctuations (see below). The above listed parameter values for κ_b, σ, ϵ and κ_P constitute the standard set of parameters values.

2 - Details of Monte Carlo algorithm

Within the Surface Evolver, we implemented finite-temperature simulations using a Monte Carlo Metropolis algorithm [22, 23] to describe the membrane dynamics. Our Monte Carlo algorithm includes four steps:
(1) Calculation of the total energy of the initial membrane configuration E_1. (2) Random selection of a vertex i_0 for a membrane fluctuation (trial move) and a vector $\mathbf{u}$ for the direction of the trial move. The lateral width w of the trial move is randomly chosen between 0 and an upper limit $w_{\max}$, possibly determined by the spacing between actin cortex-membrane linkage proteins [24]. The trial move consists in moving the vertex i_0 and all the vertices within a distance $d < w$ from i_0 by the vector $0.5\,(1+\cos(d/w))\,\mathbf{u}$. This particular function was chosen because of its regular properties at $d = w$ (vanishing of the function and its first derivative). (3) Calculation of the total energy E_2 after the trial move. (4) Decision to whether accept or reject the trial move is based on the Metropolis criterion. If $E_2 \leq E_1$, the move is accepted because the new membrane configuration has a lower energy than the initial one. If $E_2 > E_1$, the move is accepted with a probability $\exp\left[-(E_2-E_1)/(k_BT)\right]$, where T is the simulation temperature in Kelvin. If the trial move is rejected, a new trial move is selected from the same initial configuration; if it is accepted a new trial move is selected from the new membrane configuration. Between each trial move, the cell membrane vertices are examined. For the active zipper, the vertices within a cutoff distance from the particle (chosen $0.5R_0$) are immobilized, *i.e.* these vertices do not move anymore. As a consequence, the corresponding patch of membrane is stabilized, and the ligand-receptor bonds are irreversible.

For the passive zipper, none of the vertices are immobilized and all the trial moves may be reversed at a later time. Note that our choices of *simulation* parameters imply that the number of accepted and rejected fluctuations are of the same order of magnitude, which improves the convergence of the Metropolis algorithm.

Our equilibrium Monte Carlo approach is justified *a posteriori* to study the dynamics of uptake, since only a very small proportion (less than 1%) of the accepted trial moves is irreversibly immobilized. Hence, the algorithm samples membrane configurations extensively in line with equilibrium thermodynamics.

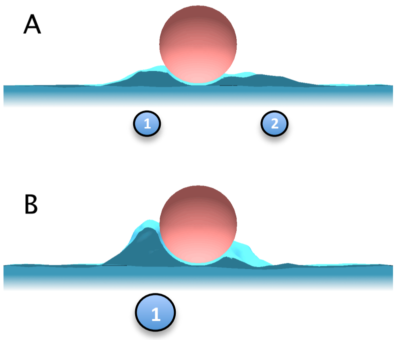

FIG. 1: Ratchet model in action. Panels (A) and (B) show phagocytic uptake at two different times with $t_A < t_B$ for the same simulation. (A) The fluctuation (1) occurs close to the particle, whereas the fluctuation (2) does not bring the cell membrane in contact with the particle. Panel (B) shows that the membrane at position (1) has progressed around the particle, whereas the membrane at position (2) has almost completely retracted to its initial flat state, demonstrating the importance of membrane immobilization by effective actin polymerization during uptake.

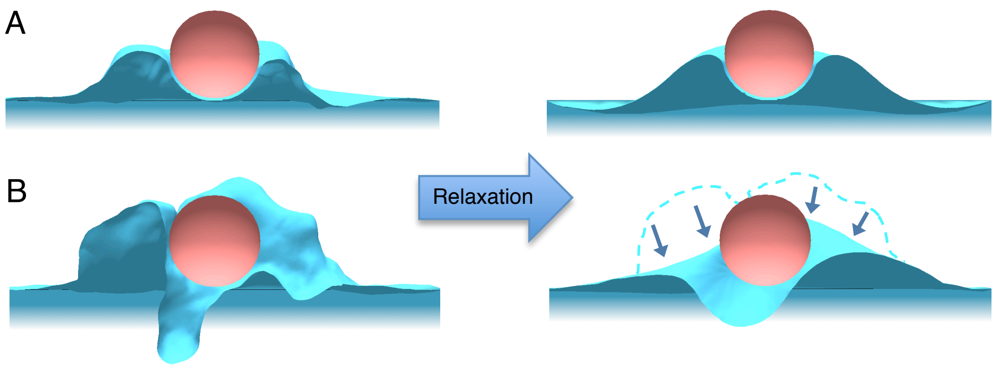

FIG. 2: Relaxation of phagocytic cup to the *ground state*. (A) Phagocytic cup shape at half-engulfment obtained for the active zipper with the set of Standard Parameters (*left*); cup relaxed at zero absolute temperature (*right*). (B) Phagocytic cup shape at half-engulfment obtained for the passive zipper with standard set of parameters (*left*); cup relaxed at zero absolute temperature (*right*), leading to unzipping.

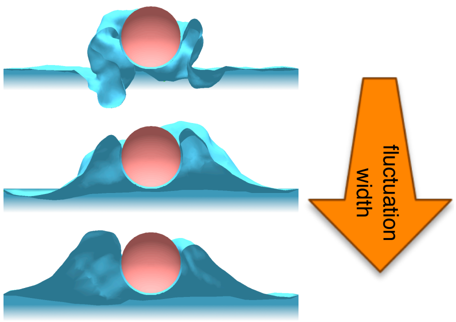

FIG. 3: Influence of the maximal width of membrane fluctuations on cup shape. Shown are (*top*) narrow fluctuations with maximal width $w_{\max} = 1.5R$, (*middle*) medium wide fluctuations with $w_{\max} = 3R$, and (*bottom*) wide fluctuations with $w_{\max} = 4R$. Parameter R is the particle radius.

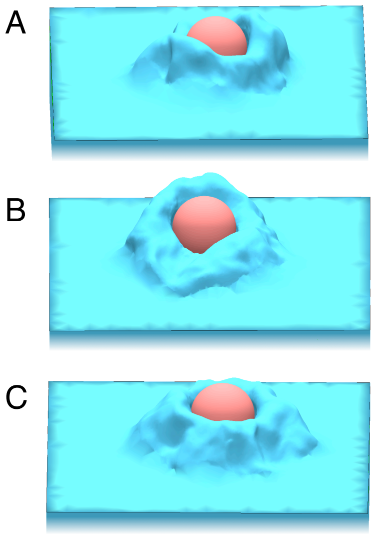

FIG. 4: Reproductibility of cup shapes for simulations under the same conditions. Side views of three simulations with the set of Standard Parameters (for approximately 50% engulfment).

Additional simulations

3 - Ratchet model in action

Our ratchet-like biophysical model is based on effective actin polymerization to render ligand-receptor bonds irreversible. As ligand-receptor binding and hence signaling can only occur in the immediate neighborhood of the particle, random membrane fluctuations far from the particle may retract with increasing simulation time. **Figure S1** gives an example of a reinforced membrane fluctuation near the particle and a vanishing membrane fluctuation further away from the particle, illustrating how this concept is implemented in the simulations.

4 - Role of engulfment kinetics in cup shape

Our simulations show that cup shape depends on the kinetics of phagocytic uptake. To demonstrate this we relaxed a phagocytic cup obtained with a finite-temperature Monte Carlo simulation towards the *ground state*, *i.e.* the shape at zero absolute temperature in the absence of thermal fluctuations (see **Figure S2**). During relaxation, the membrane evolves towards the minimal energy configuration (*right*) starting from the initial configuration (*left*). In the case of the active zipper (panel A), the resulting cup not only smoothes, but also changes shape (here it becomes broader). For other parameter choices, simulated cups are either broader or thinner than predicted by their *ground state* due to finite temperature stochastic engulfment, emphasizing the importance of the randomness of membrane fluctuations in shaping the cup.

In the case of the passive zipper (panel B), finite temperature stochastic processes are even more crucial for engulfment. Indeed, **Figure S2B** (*left*) is obtained with a simulation of the passive zipper at finite temperature. After some time, the simulation is stopped and restarted at zero absolute tempera-

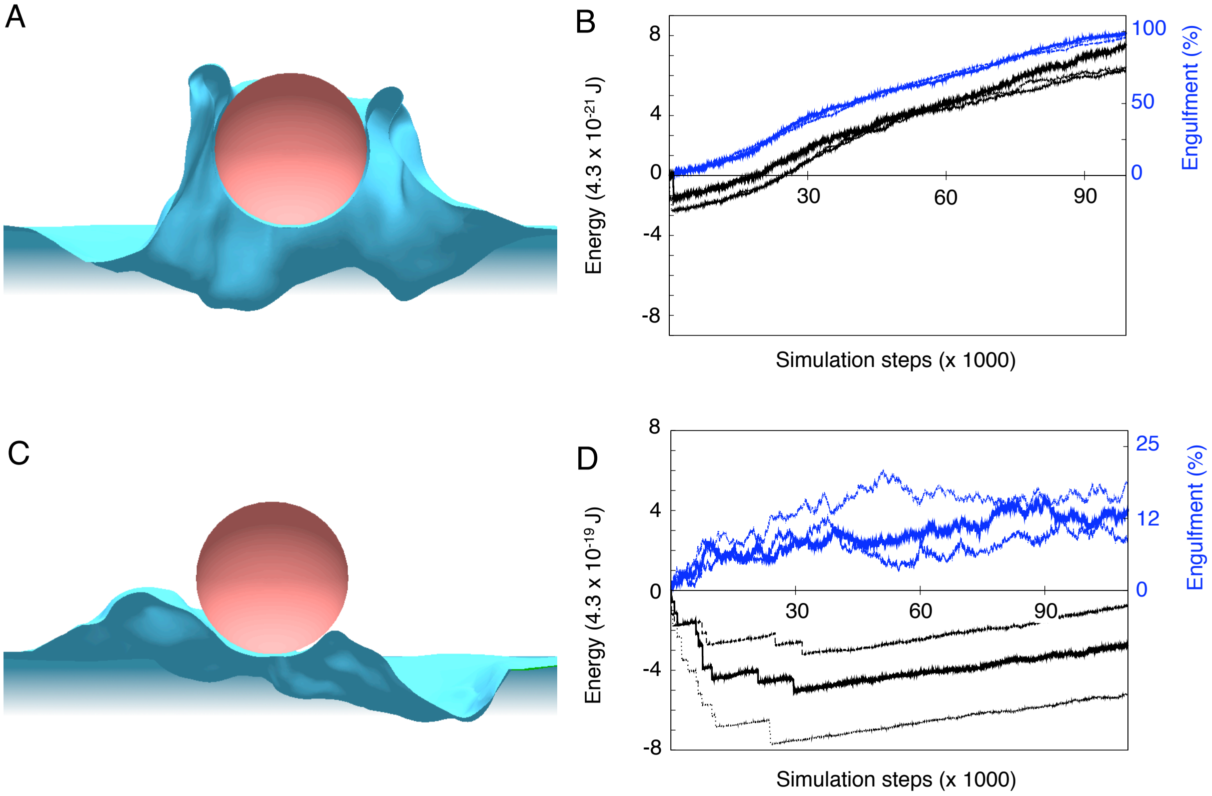

FIG. 5: Active versus passive engulfment of a large particle. (A) Cross section of phagocytic cup obtained for the active zipper. (B) Time course of total energy (black) and percentage of engulfment (blue). (C) Cross section of phagocytic cup obtained for the passive zipper (same overall simulation time). (D) Time course of total energy (black) and engulfment (blue) for the passive zipper. The particle radius is $R = 3\mu$m. Three simulations were carried out in (B,D).

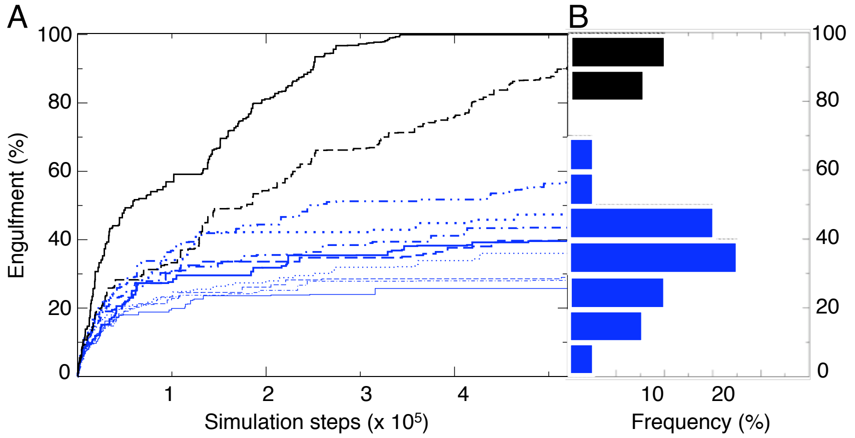

FIG. 6: Stalling at partial engulfment. (A) Time course of the percentage of engulfment as a function of increasing surface tension in simulations. Shown are completed (or nearly completed) uptake (black lines) and partial (stalled or very slowly progressing) uptake (blue lines). Surface tension values ($\sigma \times 10^{-4}$ mNm^{-1}) used are 0.8 (black solid), 1.1 (black dashed), 1.4 (thick blue dash-double dotted), 1.6 (thick blue dotted), 1.9 (thick blue dashed-dotted), 2.1 (thick blue dashed), 2.3 (thick blue solid), 2.8 (thin blue dotted), 3.1 (thin blue dash-dotted), 3.4 (thin blue dashed), and 3.7 (thin blue solid). (B) Distribution of engulfed particle surface area measured in our experiments for COS-7 cells transfected with wild-type receptors (WT-FcγR) after 10min. Black and blue bars correspond to the two peaks of the bimodal distribution.

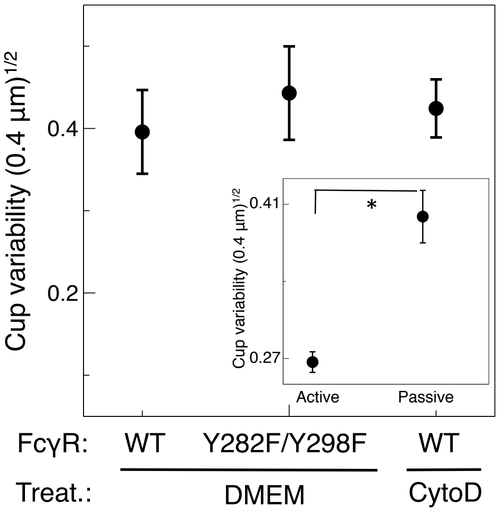

FIG. 7: Statistical analysis of phagocytic cup shapes. Cup variability (for 60 to 100% engulfed particles) was determined for COS-7 cells transfected with wild-type FcγR (WT-FcγR) or signaling-dead mutant (Y282F/Y298F-FcγR), as well as WT-FcγR cells treated with 0.2μM cytochalasin D (WT-FcγR+CytoD). The data points represent the variability averaged over all the phagocytic cups measured for given condition and range of uptake. Error bars represent the statistical standard error of the cup variability. (*Insets*) Theoretical cup variability calculated from corresponding model simulations. Student's t-test (*): p-value is less than 0.0001.

ture for relaxation of the partially engulfed state towards the *ground state*. After partial relaxation, the cup presented in **Figure S2B** (*right*) is obtained. The membrane smoothes similar to the active zipper, but also retracts, resulting in unzipping. This indicates that the release of ligand-receptor binding energy is not sufficient for uptake by the passive zipper. Additional thermal membrane fluctuations are also required. Further relaxation eventually leads to a total retraction of the cell membrane for the passive zipper.

5 - Influence of membrane fluctuation width on cup shape

The maximal width of the fluctuations $w_{\max}$ is chosen to optimize the convergence of the Monte-Carlo Metropolis algorithm (see *Methods* in the main text). This algorithm accurately samples the probability distribution of the membrane configuration, and consequently of cup shapes at different stages of uptake. The convergence of the algorithm is optimal if about half of the randomly selected membrane fluctuations (trial moves) are accepted. This condition is fulfilled for intermediate values of $w_{\max}$. **Figure S3** shows how the choice of $w_{\max}$ influences the cup shape. If $w_{\max}$ is small, only laterally small membrane fluctuations are generated by the algorithm. These fluctuations do not change the surface area and cell volume significantly, and consequently are frequently accepted by the algorithm. Hence, the cup grows fast and thin (*top* panel). In contrast, if $w_{\max}$ is large, laterally wide fluctuations may be selected, and are less frequently accepted since they can lead to drastic changes is cell volume and/or area. The fluctuation-acceptance rate is decreased, the cup grows more slowly and becomes broad (*bottom* panel).

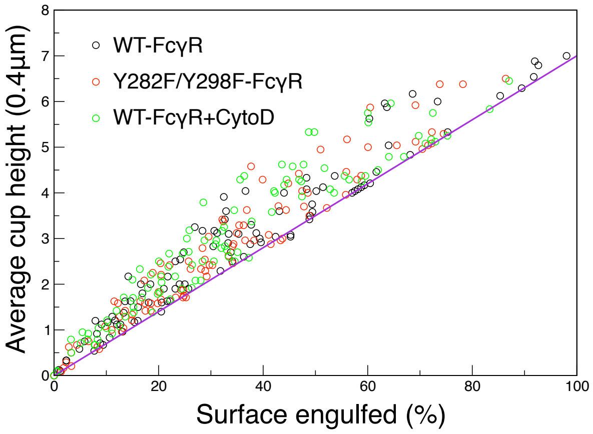

FIG. 8: Average cup height $< h >$ versus percentage of engulfed particle surface area S. Each circle corresponds to an imaged cup (black for WT-FcγR, green for Y282F/Y298F-FcγR, and red for WT-FcγR+CytoD). For a perfectly regular cup, both quantities are related by $S(\%) = 100 < h > (\mu\text{m})/(7 \times 0.4)$. Due to shape variability and finite number of angular segments used, values obtained lie slightly above the diagonal straight line.

6 - Reproductibility of cup shapes

Phagocytic cup shape is variable, in particular without the support of the actin cytoskeleton for the passive zipper. However, even for the active zipper, engulfment is inherently stochastic due to random membrane fluctuations. As depicted in **Figure S4**, simulations with the same set of parameters lead to slightly different cups after the same simulation time. This illustrates particle-to-particle variation.

7 - Active versus passive engulfment of large particles

In the main text we demonstrate that the passive zipper is able to take up small particles of radius 1.5μm, although slower and in a more variable fashion than the active zipper. Here, we compare the ability of the two zippers to take up a larger particle of radius 3μm. **Figure S5** shows that the passive zipper only engulfs a few percent of the particle surface area (panels C and D), whereas the active zipper completes uptake (panels A and B). This demonstrates that, for the uptake of large particles, ligand-receptor binding in conjunction with thermal fluctuations is not sufficient, and that the functionality of the actin network is fully required.

8 - Stalling at partial engulfment

A model previously published by one of the authors uses the force generated by actin on the cell membrane as a parameter,

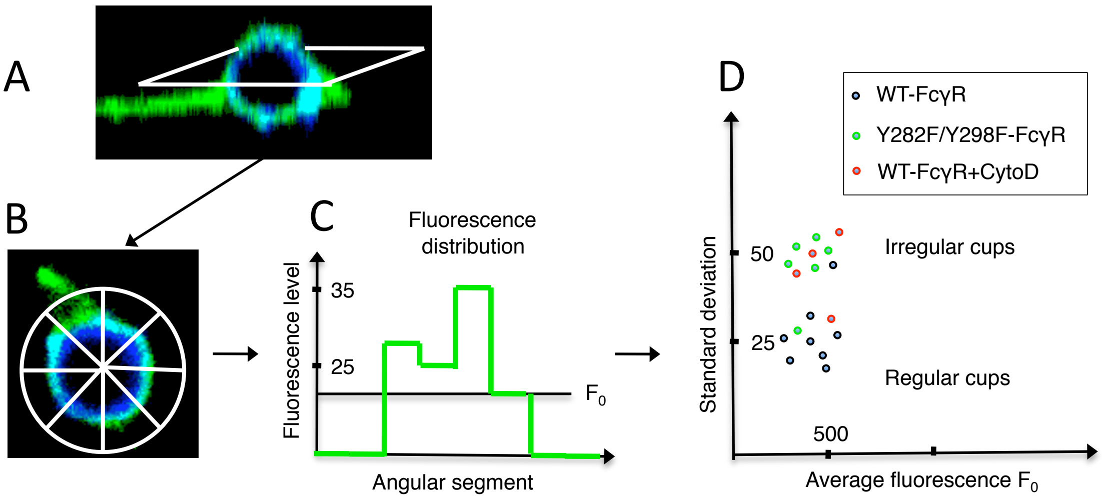

FIG. 9: Alternative image analysis of phagocytic cups. (A) Side view of a typical 3-dimensional fluorescence image, reconstructed from confocal microscopy data. Shown are the Fcγ-GFP receptors (green) and IgG ligand coating the particle (blue). The analysis plane (white square) intersects the particle at the equator. (B) Top view of the analysis plane. The green light distribution is determined using the total green light intensity in the different angular segments. (C) An example of such a distribution for one particular particle, used for extracting the average intensity I_0 and the mean-square deviation δI. (D) Illustrative scatter plot and classification of particles with respect to their engulfment statistics I_0 (horizontal axis) and normalized root mean-square deviation $\delta I_{\mathrm{N}} = \delta I/\sqrt{I_0}$ (vertical axis). Regular cups correspond to a smaller root mean-square deviation of the fluorescence distribution than variable cups.

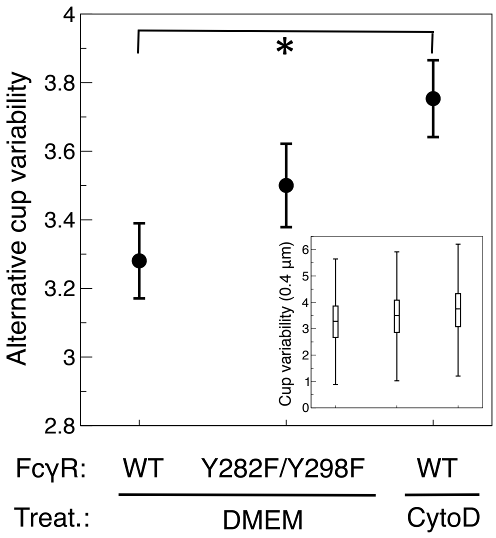

FIG. 10: Alternative statistical analysis of phagocytic cup shapes. Cup alternative variability (arbitrary units) for 30 to 70% engulfed particles, for conditions WT-FcγR, Y282F/Y298F-FcγR, and WT-FcγR+CytoD. The data points represent the variability averaged over all the phagocytic cups measured for given condition and range of uptake. Error bars represent the statistical standard error of the cup variability. Student's t-test: p-values is 0.003 (*). (*Insets*) Whisker plot representation of the cup-shape alternative variability.

and predicts that slightly different force strengths lead to very different behaviors of the phagocytic cup [25]. Specifically, a bimodal distribution of phagocytic cups was found, *i.e.* cups either fully take up the particle or stall before 50% engulfment. This was interpreted as a mechanical bottleneck at half-engulfment (see main text). Our simulations also exhibit this effect. **Figure S6A** shows how slight (much less than an order of magnitude) variations in biophysical parameters (here surface tension) may have drastic effects on the completion of uptake. This high sensitivity to small parameter changes, for specific parameter ranges, explains the particle-to-particle variation observed in our experiments (**Figure S6B**).

Additional analysis of fluorescence images

9 - Statistics of phagocytic cup shapes

In Figure 5 of the main text, we demonstrate that the cup variability in experiments is increased when signaling to the actin cytoskeleton is interrupted or when cells are treated with cytochalasin D. More precisely, this result holds at the beginning of uptake between 20 and 40% of engulfed particle area. Between 40 and 60% of the uptake, there is no significant difference between the three experimental conditions. For completeness, we report here the cup variability between 60 and 100% of uptake. **Figure S7** shows that almost completed cups are more regular (the variability is reduced by a factor of 2 compared to the range 40 to 60%), and we observe no statistically significant difference between the three different conditions. Note that the variability obtained in simulations (see **Figure S7** *inset*) is also reduced by a factor of 2, and that the relative difference in variability between the active and passive zipper is also reduced, as expected since fully

completed cups are necessarily regular.

10 - Average cup height versus percentage of engulfed particle surface area

Our image analysis method makes use of two different measures of uptake. The height h reached by the cell membrane (measured by confocal slice index), and the percentage of particle surface S covered by cell membrane. The height is used to calculate the cup variability, and the surface engulfed is used to classify the cups (20 to 40%, 40 to 60%, and 60 to 100%). **Figure S8** shows that these two quantities are indeed highly correlated. For a perfectly regular cup, the height $< h >$, averaged over angular segments, is given by $< h >= 7S/100 \times 0.4\mu$m, because each particle is imaged in 7 confocal planes. As shown, this relation does not exactly hold for variable cups, and the average height is slightly higher than predicted by the engulfed surface area. This minor deviation is due to the finite number of angular segments used.

11 - Alternative image analysis of phagocytic cup shapes

To rule out any bias in the analysis, we additionally use an alternative method to characterize cup variability. The method is based on the observation that a perfectly regular cup (at half-engulfment) reaches the equator plane all around the particle circumference, corresponding to an engulfed surface area $S = 50\%$, and an average height $< h >= 1.5\mu$m. For such a cup the distribution of FcγR-GFP fluorescence signal (indicating the presence of the cell membrane) should be uniform around the projection of the particle onto the equator plane (circle of radius R). In contrast, a variable half-engulfed cup has an irregular distribution of fluorescence intensity as the particle is only partially covered by cell membrane in its equator plane. To exploit this observation, we calculate the distribution of FcγR-GFP fluorescence along the particle's perimeter in angular segments. The standard deviation of this distribution quantifies the alternative measure of the cup variability, illustrated in **Figure S9**.

Figure S10 shows the result from the statistical analysis of phagocytic cup shapes as obtained with the alternative method. Although this analysis method is more restrictive and inaccurate, it confirms our result from the main text. Using 30 to 70% engulfed particles we obtain that cells transfected with wild-type FcγR produce significantly less variable cups than cytochalasin D treated cells (Student's t-test: p-value= 0.003).

[1] Zhelev DV, Needham D, Hochmuth RM: **Role of the membrane cortex in neutrophil deformation in small pipets.** *Biophys J* 1994, **67**: 696-705.

[2] Herant M, Heinrich V, Dembo M: **Mechanics of neutrophils phagocytosis: behavior of the cortical tension.** *J Cell Sci* 2005, **118**: 1789-1797.

[3] Herant M, Heinrich V, Dembo M: **Mechanics of neutrophils phagocytosis: experiments and quantitative models.** *J Cell Sci* 2006, **119**: 1903-1913.

[4] Charras GT, Yarrow JC, Horton MA, Mahadevan L, Mitchison TJ: **Non-equilibration of hydrostatic pressure in blebbing cells.** *Nature* 2005, **435**: 365-369.

[5] Huang H, Sylvan J, Jonas M, Barresi R, So PTC, Campbell KP, Lee RT: **Cell stiffness and receptors: evidence for cytoskeletal subnetworks.** *Am J Physiol Cell Physiol* 2005, **288**: C72-C80.

[6] Roduit C, Van der Goot FG, Los Rios P, Yersin A, Steiner P, Dietler G, Catsicas S, Lafont F, Kasas S: **Elastic membrane heterogeneity of living cells revealed by stiff nanoscale membrane domains.** *Biophys J* 2008, **94**: 1521-1532.

[7] Swanson JA, Hoppe AD: **The coordination of signaling during Fc receptor-mediated phagocytosis.** *J Leucoc Biol* 2004, **76**: 1093-1103.

[8] Gordon AH, D'Arcy Hart P, Young MR: **Ammonia inhibits phagosome-lysosome fusion in macrophages.** *Nature* 1999, **286**: 79-80.

[9] Greenberg S: **Modular components of phagocytosis.** *J Leukoc Biol* 1999, **66**: 712-717.

[10] Groves A, Dart AE, Covarelli V, Caron E: **Molecular mechanisms of phagocytic uptake in mammalian cells.** *Cell Mol Life Sci* 2008, **65**: 1957-1976.

[11] Tardieu I, Webster P, Ravesloot J, Boron W, Lunn JA, Heuser JE, Andrews NW: **Lysosome recruitment and fusion are early events required for trypanosome invasion of mammalian cells.** *Cell* 1992, **71**: 1117-1130.

[12] Hallett MB, Dewitt S: **Ironing out the wrinkles of neutrophil phagocytosis.** *Trends Cell Biol* 2007, **17**: 209-214.

[13] Phillips DJ, Wells TW, Reimer CB: **Estimation of association constants of 42 monoclonal antibodies to human IgG epitopes using a fluorescent sequential-saturation assay.** *Immunol Lett* 1987, **17**: 159-168.

[14] Raychaudhuri G, McCool D, Painter RH: **Human IgG1 and its Fc fragment bind with different affinities to the Fc receptors on the U937, HL-60, and ML-1 cell lines.** *Mol Immunol* 1985, **22**: 1009-1019.

[15] Wallace PK, Keler T, Coleman K, Fisher J, Vitale L, Graziano RF, Guyre PM, Fanger MW: **Humanized mAb H22 binds the human high affinity Fc receptor for IgG (Fcγ RI), blocks phagocytosis, and modulates receptor expression.** *J Leukoc Biol* 1997, **62**: 469-479.

[16] Keown MB, Henry AJ, Ghirlando R, Sutton BJ, Gould HJ: **Thermodynamics of the interaction of human immunoglobulin E with its high affinity receptor Fcϵ RI.** *Biochemistry* 1998, **37**: 8863-8869.

[17] Gandour DM, Walker WS: **Macrophage cell cycling: influence on Fc receptors and antibody-dependent phagocytosis.** *J Immunol* 1983, **130**: 1008-1012.

[18] Morris CE, Homann U: **Cell surface area regulation**

and membrane tension. *J Membrane Biol* 2001, **179**: 79-102.

[19] Wehner F, Olsen H, Tinel H, Kinne-Saffran E, Kinne RKH: **Cell volume regulation: osmolytes, osmolyte transport, and signal transduction.** *Rev Physiol Biochem Pharmacol* 2003, **148**: 1-80.

[20] Moseley JB, Mayeux A, Paoletti A, Nurse P: **A spatial gradient coordinates cell size and mitotic entry in fission yeast.** *Nature* 2009, **459**: 857-860.

[21] Tzur A, Kafri R, LeBleu VS, Lahav G, Kirschner MW: **Cell Growth and Size Homeostasis in Proliferating Animal Cells.** *Science* 2009, **325**: 167-171.

[22] Metropolis N, Rosenbluth AW, Rosenbluth MN, Teller AH, Teller E: **Equations of State Calculations by Fast Computing Machines.** *J Chem Phys* 1953, **21**: 1087-1092.

[23] Piotto S, Mavelli F: **Monte Carlo simulations of vesicles and fluid membranes transformations.** *Ori Life Evo Bios* 2004, **34**: 225-235.

[24] Charras GT, Hu CK, Coughlin M, Mitchison TJ: **Reassembly of contractile actin cortex in cell blebs.** *J Cell Biol* 2006, **175**: 477-490.

[25] Van Zon JS, Tzircotis G, Caron E, and Howard M: **A mechanical bottleneck explains the variation in cup growth during FcγR phagocytosis.** *Mol Sys Biol* 2009, **5**: 298.
